# The protective effect of *Nigella sativa* against cisplatin-induced nephrotoxicity in rats

**Published:** 2016

**Authors:** Sara Hosseinian, Abolfazl Khajavi Rad, Mousa-Al-Reza Hadjzadeh, Nema Mohamadian Roshan, Shahrzad Havakhah, Somayeh Shafiee

**Affiliations:** 1 *Department of Physiology, School of Medicine, Mashhad University of Medical Sciences, Mashhad, Iran*; 2 *Neurogenic Inflammation Research Center, Department of Physiology, School of Medicine, Mashhad University of Medical Sciences, Mashhad, Iran*; 3 *Neurocognitive Research Center, Department of Physiology, School of Medicine, Mashhad University of Medical Sciences, Mashhad, Iran*; 4 *Departmant of Pathology, Qaem Hospital, Mashhad University of Medical Sciences, Mashhad, Iran*

**Keywords:** *Cisplatin*, *Nigella sativa*, *Vitamin E*, *Renal Failure*

## Abstract

**Objective:**

The clinical use of cisplatin is highly restricted, because of its nephrotoxicity. In this study the protective effect of *Nigella sativa* (*N. sativa*) against cisplatin-induced nephrotoxicity was investigated in rats.

**Materials and Methods:**

In the current study, the effects of the administration of aqueous-ethanolic extract of *N. sativa *(100 and 200 mg/kg, BW) and vitamin E (100 mg/kg, BW) against blood and urine biochemical alterations and kidney function in rats treated with cisplatin were investigated. Cisplatin was injected at a dose of 6 mg/kg, BW, on the sixth day of the experiment.

**Results:**

The results indicated significant changes in serum urea and creatinine concentration, urine glucose concentration, and urine output in cisplatin group compared with control group. Serum urea and creatinine concentration in preventive and preventive+treatment vitamin E and preventive+treatment *N. sativa* (200 mg/kg, BW) groups and also serum creatinine concentration in preventive+treatment *N. sativa* (100 mg/kg, BW) group significantly decreased compared with cisplatin group. Urine glucose concentration in preventive and preventive+treatment *N. sativa* groups and urine output in preventive and preventive+treatment *N. sativa* (200 mg/kg, BW) groups significantly decreased compared with cisplatin group.Osmolarity excretion rate in preventive and preventive+treatment vitamin E and preventive *N. sativa* groups was significantly higher than control group.

**Conclusions:**

The current study suggests that *N. sativa* extract and vitamin E in a dose- and time-dependent manner improved the serum and urine biochemical parameters and kidney function in cisplatin-induced nephrotoxicity in rats. However, it needs more investigations to determine the mechanism of *N. sativa* action on cisplatin-induced kidney toxicity.

## Introduction

Cisplatin is one of the important anti-neoplastic drugs that is useful in the treatment of many tumors including head and neck, ovary, testis, and lung malignancies (Katzung, 2004 ▶). Therapeutic effects of cisplatin are associated with severe side effects, mainly nephrotoxicity and neurotoxicity (Ito et al., 1998 ▶; Yao et al., 2007 ▶). Cisplatin therapeutic action is dose-dependent. However, the major restriction to use a high dose of cisplatin is its strong side effects in the kidney and gastrointestinal tract (Razzaque, 2007 ▶). The highest concentration of cisplatin accumulates in S3 segment of the proximal tubule followed by the distal collecting tubule and the S1 segment of the proximal convoluted tubule (Kroning et al., 2000 ▶). 

Original pathway of cisplatin transport in renal cells is active transport, although cisplatin enters the cells through passive diffusion as well. The organic cation transporter 2 (OCT2) is the major transporter for cisplatin uptake in proximal tubular cells (Ciarimboli, 2014 ▶). Intracellular effects of cisplatin include reduction in natural activity of ATPase, mitochondrial damage, cell cycle arrest, and disturbance in cellular transport systems. Sum of these effects can induce apoptosis or necrosis (Chirino and Pedraza-Chaverri, 2009 ▶). 

Cisplatin also induces the production of free radicals and activates the mitogen-activated protein kinase (MAPK) intracellular signaling pathways (Arany et al., 2004 ▶). In the presence of cisplatin, reactive oxygen species (ROS) are generated in cells via the xanthine-xanthine oxidase system, mitochondria, and NADPH oxidase (Kawai et al., 2006 ▶). Inflammation which is mainly induced via TNF-α production has an important role in pathogenesis of cisplatin-induced nephrotoxicity (Ramesh and Reeves, 2004 ▶). 

All of these factors cause tubular damage and dysfunction. In this regard, there is an increasing interest in finding new remedies to minimize the cisplatin-induced nephrotoxicity. *Nigella sativa* Linn. (*N. sativa*) belongs to family Ranunculaceae is a herbaceous annual plant that commonly known as black cumin and black seed (Butt and Sultan, 2010 ▶). *N. sativa* has been traditionally used in India, Europe, Middle East, Far East, and South-East Asia as spices and natural remedy for several ailments including asthma, headache, infections, obesity, fever, vertigo, hypertension, influenza, and cough (Ali and Blunden, 2003 ▶; Salem, 2005 ▶). There is also an Islamic belief that black cumin is a remedy for all illnesses, except ageing and death (Randhawa, 2008 ▶). *N. sativa* major chemical components are 36-38% fixed oil, 0.4-2.5% essential oil, alkaloid, saponin, mineral elements, proteins, vitamins, and carbohydrates (Salem, 2005 ▶; Randhawa, 2008 ▶; Al-Naghib et al., 2009 ▶). 

It has been reported that *N. sativa* exhibits many pharmacological effects, including antioxidant (Ashraf et al., 2011 ▶), anti-inflammatory (Chehl et al., 2009 ▶), antimicrobial (Morci, 2000 ▶), antidiabetic (Alimohammadi et al., 2013 ▶), antihypertensive (Fallah Huseini et al., 2013 ▶), Neuroprotective (Khazdair, 2015 ▶), and anticarcinogenic (Khan et al., 2011 ▶) properties. It has also been shown that *N. sativa* seeds and/or its constituents have protective effects against nephrotoxins. Yaman and Balikci reported that *N. sativa* oil (0.2 ml/kg) protected the rats from gentamicin-induced nephrotoxicity (Yaman and Balikci, 2009). Furthermore, Badary et al. (1997) ▶ showed that oral administration of thymoquinone (TQ), 5 days before and 5 days after of cisplatin injection protected against nephrotoxic effect of cisplatin in rats and mice (Badary et al., 1997 ▶). 

Therefore, the aim of this study was to investigate the effects of *N. sativa* seeds on cisplatin-induced nephrotoxicity in rat.

## Materials and Methods

**Chemicals**

Cisplatin was purchased from the Mylan Company (Greece). *N. sativa* seeds were obtained from the local market and was identified by botanists in the herbarium of Ferdowsi University of Mashhad with herbarium number 293-0303-1. Urea, creatinine, and glucose kits were obtained from the Pars Azmoon Company (Tehran, Iran).

**Extract Preparation**

For the preparation of the hydroalcoholic extract, 50 g of the powdered seeds was extracted with 500 mL ethanol (70%, v/v). After the extraction, the solution was purified using a rotary vacuum evaporator which yielded a blackish-brown concentrate. The prepared extract was kept at 4 ˚C prior to use. 

**Animals**

Eighty male Wistar Albino rats weighing 230-300 g obtained from the Animal House of the School of Medicine, Mashhad University of Medical Sciences. The rats were housed at 23±2 ^˚^C with a relative 50-60% humidity and a 12:12h light-dark cycle and free access to standard laboratory food and water. All experiments were carried out under the authority of the Mashhad University of Medical Sciences and the norms of international animal ethics were followed.

**Experimental Design**

In this study, the rats were randomly divided into 8 groups of 10 each:

Control group: received normal saline (i. p.) for 11 consecutive days. 

Cisplatin group: received normal saline (i. p.) for 11 consecutive days and cisplatin (6 mg/kg BW, i. p.) on the sixth day of experiment.

Preventive vitamin E group: received vitamin E (100 mg/kg BW, i. p.) for 6 consecutive days and cisplatin (6 mg/kg BW, i. p.) on the sixth day of experiment.

Preventive* N. sativa* groups: received *N. sativa* extract (100 and 200 mg/kg BW, i. p.) for 6 consecutive days and cisplatin (6 mg/kg BW, i. p.) on the sixth day of experiment.

Preventive+treatment vitamin E group: received vitamin E (100 mg/kg BW, i. p.) for 11 consecutive days and cisplatin (6 mg/kg BW, i. p.) on the sixth day of experiment.

Preventive+treatment *N. sativa* groups: received *N. sativa* extract (100 and 200 mg/kg BW, i. p.) for 11 consecutive days and cisplatin (6 mg/kg BW, i. p.) on the sixth day of experiment.

24-hour urine samples were collected on the 0 and 12^th^ days of the study, while each animal was housed in a separate metabolic cage. Blood was also collected from the orbital sinus on days 0 and 12. Then, all animals were humanely sacrificed on the 12^th^ day of the experiment. Blood samples were centrifuged at 4000 g for 10 min, and serum was stored at -20 ˚C until assayed. Serum urea and creatinine concentration as well as urine glucose concentration were measured by *Convergys*^®^* 100 Biochemistry Analyser*using commercial kits (Pars Azmoon Company, Tehran, Iran). Urine osmolarity was determined on a cryoscopicosmometer (*Osmomat*^®^* 030*).

**Statistical analysis**

The data were expressed as means±SEM. For each data group, the differences between days 12 and 0 were calculated. Homogeneity of variance was tested using Levene's test. Differences between group means were estimated using one-way analysis of variance (ANOVA) followed by LSD test for multiple comparisons. A p value less than 0.05 was considered as significant.

## Results

**Figure 1:**
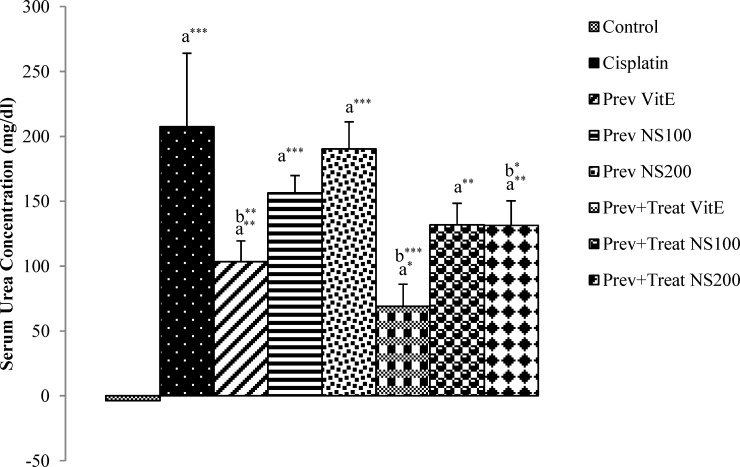
Difference of serum urea concentration between 12^th^ and 0 days in all experimental groups of animals. Values are the mean±SEM. The data were analyzed using one-way ANOVA and post hoc LSD. A significant difference was considered at p<0.05. *p<0.05, ** p<0.01, *** p<0.001.a: significant difference from control group, b: significant difference from cisplatin group

**Figure 2:**
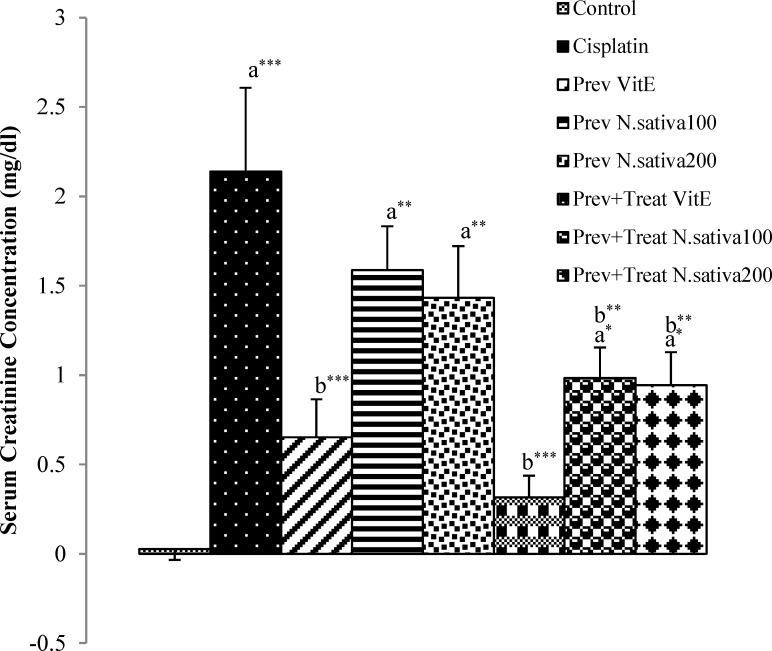
Difference of serum creatinine concentration between 12^th^ and 0 days in all experimental groups of animals. Values are the mean±SEM. The data were analyzed using one-way ANOVA and post hoc LSD. A significant difference was considered at p<0.05. *p<0.05, ** p<0.01,*** p<0.001. a: significant difference from control group, b: significant difference from cisplatin group

Serum urea and creatinine concentration in cisplatin group demonstrated a significant raise compared with the control animals (p<0.001) (Figures 1, 2). However, both of these markers in preventive and preventive+treatment vitamin E (p<0.01 and p<0.001, respectively) and in preventive+treatment *N. sativa* (200 mg/kg BW) (p<0.01 and p<0.05, respectively) groups were significantly lower than cisplatin group (Figures 1, 2). There were also a significant decrease (p<0.01) in serum creatinine level in preventive+treatment *N. sativa* (100 mg/kg BW) group compared with cisplatin group (Figure 2).

Compared to cisplatin group, the reduction of serum urea concentration in preventive and preventive+treatment *N. sativa* (100 mg/kg, BW) groups was 24.5% and 36.4% (p=0.15 and p=0.06, respectively) and the decrease of serum creatinine level in preventive *N. sativa* (100 and 200 mg/kg, BW) groups was 25.8% and 32.86% (p=0.09 and p=0.05, respectively), but these reductions was not significant (Figures 1 and 2).

**Figure 3:**
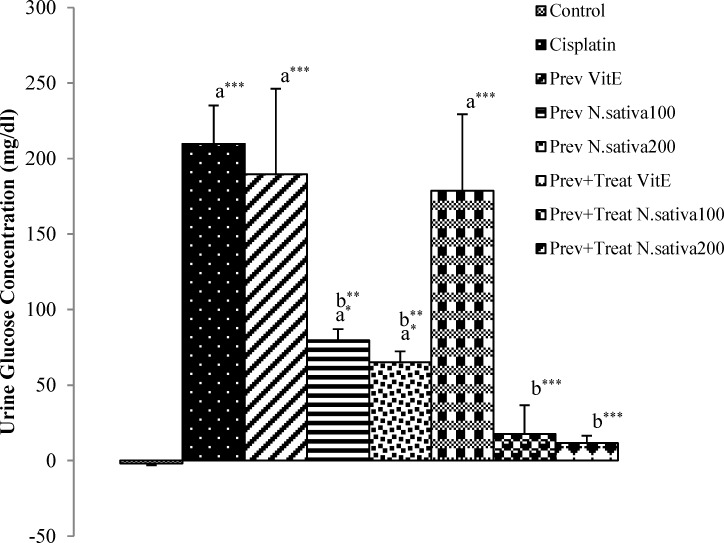
Difference of urine glucose concentration between 12^th^ and 0 days in all experimental groups of animals. Values are the mean±SEM. The data were analyzed using one-way ANOVA and post hoc LSD. A significant difference was considered at p<0.05. *p<0.05, ** p<0.01,*** p<0.001. a: significant difference from control group, b: significant difference from cisplatin group

Urine glucose concentration in cisplatin group showed a significant increase compared with the control animals (p<0.001) (Figure 3). However, there was a significant decrease in urine glucose concentration in preventive *N. sativa* (100 and 200 mg/kg, BW) and preventive+treatment *N. sativa* (100 and 200 mg/kg, BW) groups compared to cisplatin group (p<0.01 and p<0.001, respectively) (Figure 3). However, vitamin E did not show a significant effect on urine glucose concentration in the preventive and preventive+treatment groups compared with the cisplatin treated animals (Figure 3). preventive *N. sativa *(100, 200 mg/kg, BW) groups was 25.8% and 32.86% (p=0.09 and p=0.05, respectively), but these reductions was not significant (Figure. 1, 2). Urine glucose concentration in cisplatin group showed a significant increase compared with the control animals (p<0.001) (Figure 3). However, there was a significant decrease in urine glucose concentration in preventive *N. sativa *(100, 200 mg/kg, BW) and preventive+treatment *N. sativa *(100, 200 mg/kg, BW) groups compared to cisplatin group (p<0.01 and p<0.001, respectively) (Figure 3). 

**Figure 4:**
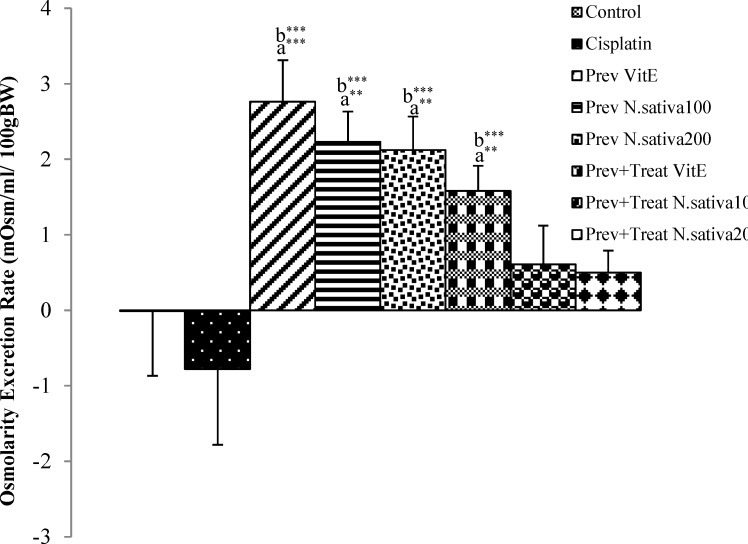
Difference of osmolarity excretion rate between 12^th^ and 0 days in all experimental groups of animals. Values are the mean±SEM. The data were analyzed using one-way ANOVA and post hoc LSD. A significant difference was considered at p<0.05. ** p<0.01,*** p<0.001*. a*: significant difference from control group, b: significant difference from cisplatin group

However, vitamin E did not show a significant action on urine glucose concentration in the preventive and preventive+treatment groups compared with the cisplatin treated animals (Figure 3). Osmolarity excretion rate in cisplatin group demonstrated no significant change compared with the control animals (Figure 4). Osmolarity excretion rate in preventive and preventive+treatment vitamin E (p<0.001) and preventive *N. sativa *(100 and 200 mg/kg, BW) (p<0.001) groups showed a significant increase compared with the control group (Figure 4). Moreover, the amount of this marker in preventive+treatment* N. sativa *(100 and 200 mg/kg, BW) groups did not show a significant difference compared with the control animals (Figure 4).

**Figure 5:**
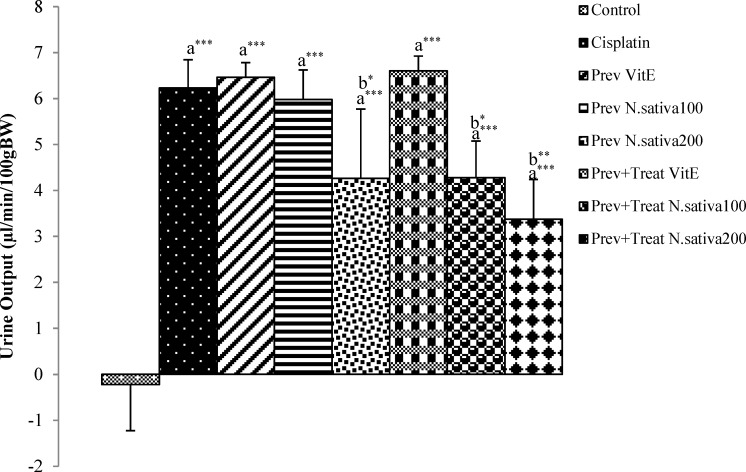
Difference of urine output between 12^th^ and 0 days in all experimental groups of animal. Values are the mean±SEM. The data were analyzed using one-way ANOVA and post hoc LSD. A significant difference was considered at p<0.05. *p<0.05, ** p<0.01,*** p<0.001. a: significant difference from control group, b: significant difference from cisplatin group

Cisplatin injection caused a significant increase of urine output in cisplatin group compared with the control group (p<0.001) (Figure 5). *N. sativa *extract administration at 100 mg/kg BW in preventive+treatment group and 200 mg/kg BW in preventive and preventive+treatment groups significantly reduced the urine output as compared with the cisplatin treated animals (p<0.05, p<0.05, and p<0.01, respectively) (Figure 5). However, vitamin E administration in preventive and preventive+treatment groups was not able to reduce urine output compared with the cisplatin group (Figure 5).

## Discussion

Nephrotoxicity induced by cisplatin as an important antineoplastic drug is one of the prominent causes of acute renal injury (Yao et al., 2007 ▶). Various studies have shown that cisplatin induces apoptosis or necrosis in the kidney cells by reduction in ATPase activity and induction of cell cycle arrest and also disruption of renal tubular cell transport systems (Razzaque, 2007 ▶). Other mechanisms of cisplatin-induced kidney injury involve inflammation, fibrogenesis, and oxidative stress (Yao et al., 2007 ▶). In recent years, use of natural remedies has developed to reduce cisplatin nephrotoxic action (Antunes et al., 2001 ▶; Kim et al., 2006 ▶; Shimeda et al., 2005 ▶). Among medicinal plants, *N. sativa *because of various pharmacologic properties and rich historical and religious background, takes into more consideration (Ahmad et al., 2013 ▶).

Therefore, in the present study we hypothesized that *N. sativa *extract may protect the kidney against cisplatin-induced toxicity. Present study, in the line with Salama et al. (2011) ▶ and Tikoo et al. (2007) ▶ observations, demonstrated that cisplatin was significantly able to increase serum urea and creatinine concentration, which confirmed the accuracy of the experiment in induction of renal injury (Tikoo et al., 2007 ▶; Salama et al., 2011 ▶). The results of current study indicated that treatment with *N. sativa *extract at 200 mg/kg BW significantly reduced the serum urea and creatinine concentrations in preventive+treatment group. Moreover, administration of *N. sativa *extract at 100 mg/kg BW in preventive+treatment group decreased the serum creatinine concentration significantly. These results are in accordance with previous studies reporting that oral administration of thymoquinone 5 days before and 5 days after the cisplatin injection reduced the cisplatin-induced elevation of serum urea and creatinine concentration (Badary et al., 1997 ▶). Moreover, Salama et al. (2011) ▶ and El-Daly (1996) ▶ described that administration of *N. sativa *extract started before and concomitant with alternative injections of cisplatin decreased the cisplatin-induced elevation of serum urea and creatinine concentration (El Daly, 1997 ▶; Salama et al., 2011 ▶). There are evidences indicating that cisplatin exerts its enhancing effect in serum urea and creatinine concentration by the reduction of glomerular filtration rate (GFR) that may be due to production of reactive oxygen species (ROS) in afferent and efferent arterioles and consequent increase in their resistance. ROS also increase the production of vasoconstrictor substances including endothelin, isoprostane, and thromboxane that in turn result in glomerular vasoconstriction and GFR reduction (Nath and Norby, 2000 ▶; Miller et al., 2010 ▶). On the same basis, the protective effect of *N. sativa *extract may be related to its antioxidant and cytoprotective effects (Ali and Blunden, 2003 ▶; Ashraf et al., 2011 ▶). Moreover, present results revealed that the beneficial action of *N. sativa *on serum urea and creatinine concentration in preventive+treatment *N. sativa *groups was more than preventive *N. sativa *groups, which might be due to time effect of *N. sativa *administration on the kidney injury amendment. This finding is in agreement with Rooney et al. (2005) ▶ and Alimohammadi et al. (2013) ▶ that reported the time dependency of antidiabetic and anticancer effects of *N. sativa *(Rooney and Ryan, 2005 ▶; Alimohammadi et al., 2013 ▶). In addition, enhanced urine glucose concentration followed by cisplatin injection in the current study was also reported by Portilla et al. (2006) ▶ and Naghizadeh et al. (2008) ▶ (Portilla et al., 2006 ▶; Naghizadeh et al., 2008 ▶). In the present work, administration of *N. sativa *extract at 100 and 200 mg/kg BW in preventive and preventive+treatment groups significantly decreased the urine glucose concentration. Present study is in agreement with Badary (1999) ▶ that showed the regressive effect of thymoquinone on the ifosfamide-induced elevation of urine glucose concentration (Badary, 1999 ▶). The glycosuria induced by cisplatin administration is possibly due to direct inhibition of sodium-glucose transporters (SGLT) in tubular epithelial cells via the covalent platinum binding to SH groups of these transporters. Another possible mechanism involved is direct inhibition of Na^+^-K^+ ^ATPase pump in basolateral membrane of proximal tubular epithelial cells and also inhibition of ATP synthesis by interference of cisplatin with mitochondrial function. *N. sativa* extract probably through its antioxidant effect improves the mitochondrial function and increases the ATP production (Kim et al., 1995 ▶; Ikari et al., 2005 ▶; Egawa-Takata et al., 2010 ▶). In the present study, cisplatin caused no significant change in osmolarity excretion rate compared with the control group and *N. sativa* extract and vitamin E administration led to a significant increase in osmolarity excretion rate in preventive *N. sativa* (100 and 200 mg/kg, BW) and preventive and preventive+treatment vitamin E groups compared with the control group. Osmolarity excretion rate in preventive+treatment *N. sativa *(100 and 200 mg/kg, BW) groups did not show a significant difference compared with the control animals. The exact mechanism of cisplatin effect on osmolarity excretion rate has not been fully elucidated but it seems that decrease in gene expression or dysfunction of sodium and potassium transporters as well as increase in urine output due to aquaporins dysfunction might be involved and *N. sativa *extract probably in a time-dependent manner has been able to reduce these adverse effects of cisplatin. In addition, the results of the present investigation indicated that cisplatin administration in accordance with Kishore et al. (2000) ▶ and Francescato et al. (2004) ▶ findings caused a significant increase in urine output (Kishore et al., 2000 ▶; Francescato et al., 2004) ▶. However, *N. sativa *extract at 200 mg/kg BW in preventive and preventive+treatment groups was significantly able to decrease output that is in line with Badary et al. (1997) ▶ investigation (Badary et al., 1997 ▶). The polyuria followed by cisplatin administration might be the result of disturbance in urine concentration mechanism that in turn is possibly due to defect at the level of the G protein and also reduced cAMP production that leads to resistance to vasopressin as well as decrease in AQP1 gene expression in the cells of the proximal tubule and the descending limb of Henle’s loop. Indeed, in the collecting duct principal cells, cisplatin decreases AQP2 gene expression in subapical vesicles as well as the apical membrane and AQP3 gene expression in the basolateral membrane (Wong et al., 1993 ▶; Kim et al., 2001 ▶). Although, the exact mechanism of the effect of NS extract on water transport in renal tubules is not clear, it seems that NS extract via the improvement of cAMP signaling pathway and potentiation of vasopressin function on renal tubules and aquaporins amends the cisplatin toxic action on kidney water reabsorption. Moreover, beneficial effect of *N. sativa* on tubular glucose transport is another possible reason for reduction in urine output. Furthermore, this work showed that *N. sativa* extract better than vitamin E eliminates the toxic effects of cisplatin on water transport in the kidney, which is possibly due to diverse effects of *N. sativa* extract beside its antioxidant effect.

In conclusion, the current investigation revealed that administration of *N. sativa* extract works better than vitamin E to reduce toxic effects of cisplatin in a time- and dose-dependent manner. Further investigations are required to elucidate the mechanisms of beneficial actions of *N. sativa* in cisplatin-induced nephrotoxicity.
